# Differences in Consumer Preferences for Lamb Meat before and during the Economic Crisis in Spain. Analysis and Perspectives

**DOI:** 10.3390/foods9060696

**Published:** 2020-05-29

**Authors:** Adrián Rabadán, Laura Martínez-Carrasco, Margarita Brugarolas, Casilda Navarro-Rodríguez de Vera, Estrella Sayas-Barberá, Rodolfo Bernabéu

**Affiliations:** 1 Escuela Técnica Superior de Ingenieros Agrónomos y de Montes (ETSIAM), Universidad de Castilla-La Mancha, Campus Universitario s/n, 02071 Albacete, Spain; Rodolfo.Bernabeu@uclm.es; 2 Escuela Politécnica Superior de Orihuela (EPSO), Universidad Miguel Hernández, Avenida de la Universidad de Elche, s/n, 03202 Elche, Spain; lmartinez@umh.es (L.M.-C.); mbrugaro@umh.es (M.B.); casilda.navarro@umh.es (C.N.-R.d.V.); estrella.sayas@umh.es (E.S.-B.)

**Keywords:** agro-food marketing, consumer behavior, economic crisis, food safety, origin

## Abstract

Determining the preferences of food consumers is key for adapting supply and demand. This adaptation of supply is dynamic rather than static, given that it develops over time and is influenced by both social and economic factors. This work presents an analysis of the development of lamb meat consumption at two points in time, 2004 and 2014, before and in the midst of the economic crisis in Spain (2007–2017). Our findings show that together with the external appearance and against the backdrop of an economic recession, price has a greater impact on consumers’ purchasing decisions than origin and quality seals, despite these being attributes that are traditionally used as a guarantee of food safety and traceability. This suggests that in times of economic crisis consumer preferences shift towards attributes that are less related to product quality. Nonetheless, the comparison of the consumer segments for each of the years under study revealed that age and level of education are the socioeconomic factors that most influence the preferences of lamb meat consumers.

## 1. Introduction

Broadly speaking, meat consumption has remained stable in the European Union (EU) over recent years, despite a reduction in certain consumer segments [1,2,3,4]. However, a clear trend has been observed whereby consumers are shifting from the consumption of red meat, such as beef or lamb, towards white meat, such as chicken [5], primarily as a result of the publication of numerous studies relating red meat consumption to higher mortality rates [6,7].

Lamb meat production in the EU fell 17.62% between 2007 and 2017 [8], threatening the survival of a sector which, despite its limited size, is essential to the settlement of population and generation of income in rural areas, where other economic activities are lacking [9,10,11]. Specifically, in Spain the production of lamb meat in Spain decreased from 20,329 tons in 2004 to 11,511 tons in 2014 [8]. At the same time, lamb meat prices have not changed severely [12]. Despite the large share of total livestock greenhouse gas emissions of ruminants [13], lamb meat production in Spain is traditionally developed in rural areas in which the extensive production of other types of meat would be compromised due to adverse weather conditions. Given the relevance of lamb meat production in the producing countries and the severe reduction of lamb meat consumption in some of its traditional markets, it is even more important to adapt the product to consumers’ preferences and desires. In this sense, numerous studies have analyzed the formation of consumer preferences when purchasing lamb meat [14,15,16,17,18,19,20,21].

Since meat is considered a potentially harmful product for health, correct labelling is an important factor for consumers [22]. Drawing on studies conducted in various countries, there appears to be a general consensus on the importance consumers attach to the origin of lamb meat, given its significance for levels of quality and food safety [16,23,24]. Several studies have shown that Spanish consumers prefer lamb meat produced within the country [20,24]. Similarly, a study developed about the preferences of consumers from Spain, France, and the United Kingdom reported that consumers from those countries preferred lamb meat produced nationally [23].

Regarding other quality attributes, quality labels have also been reported as a distinguishing element for meat products [25]. Bernabéu, Rabadán, El Orche, and Díaz [16] reported that the geographical label was one of the most important attributes for lamb meat consumers after meat origin and the meat type. There also seem to be specific consumer segments that when purchasing lamb meat pay special attention to other attributes, such as other quality labels [14,15,18], breed [17,26], production system [14,17], and brand [26].

Price, however, appears not to have a key role in determining preferences among lamb meat consumers. Indeed, it has been found that price has a very limited impact on the formation of consumer preferences compared to other attributes, such as the type or origin of the meat [16,20], while other studies have reported it to be the least important of the attributes taken into consideration [20,23]. These results are typically explained by reference to the fact that lamb is more expensive than other meats, and thus lamb meat consumers are less sensitive to variations than meat consumers in general. However, it is worth underlining that the average selling price of lamb meat in the EU has been falling continuously since 2011 [27].

The influence of such attributes on consumer preferences in general and on lamb meat consumers specifically have essentially been the subject of static analysis [15,16,19], with there being a lack of information on the stability of these preferences over time or when confronted by a turbulent economic environment.

Most studies coincide in finding that times of recession caused by economic crises have a notable impact on consumption, resulting in significant changes in consumption patterns [28,29,30,31]. More specifically, some studies have suggested that situations of economic crises enhance the development of healthier behaviors, and have an impact on diet [32]. During the economic crisis of 2007–2017, for example, the consumption of sweets in Iceland decreased significantly [33].

Nonetheless, not all consumer groups react to a crisis in the same way; changes emerge depending on socioeconomic characteristics and personality. The economic crisis that affected Indonesia between 1993 and 2000 had a more negative impact, for example, on consumers with a lower level of education and those resident in urban areas and provinces [31], while in the Asian economic crisis, the greatest product-related consumption adjustments were found among the most risk-adverse individuals [29].

In a study conducted in Spain, Díaz-Méndez and García-Espejo [34] found that during the 2007–2017 economic crisis the consumption gap converged downwards, regardless of income, in the case of the most expensive products and those for which medical guidelines recommend reduced intake.

Similar results for this clear trend of reduced expenditure even among the highest income groups, were also reported in the study by Kotelnikova and Radaev [35], which compared overall food consumption in Russia using data obtained between 2004 and 2014.

Price is one of the attributes whose relative importance most increases in periods of economic turmoil. In Greece, during the 2007–2017 economic crisis, the quantities of food consumed fell significantly, with consumers opting increasingly for less expensive brands [36]. The same trend towards a preference for generic products and cheaper brands was also reported during the Asian crisis [29] and the recession in Portugal resulting from the 2007–2017 crisis [37]. Specifically, during the economic crisis in Spain, it was found that consumers noticeably moved towards lower-priced retail outlets [38].

The 2007–2017 economic crisis in Spain had a major impact on consumer behaviors [30], with meat consumption declining significantly [34]. However, there is a lack of information about the specific impact on the relative importance of the attributes that form consumer preferences products.

Therefore the aim of the present work was to analyze the evolution of consumers’ preferences of lamb meat in Spain before (2004) and during (2014) the economic crisis, in order to examine the variations in the importance of the main attributes of lamb meat consumption and the applicability of these in response to future crises.

## 2. Materials and Methods

### 2.1. Participants

For this paper, a survey was conducted on 800 lamb meat consumers (400 consumers in Castillo-La Mancha in March 2004 and 400 consumers in Madrid in November and December 2014). Those surveyed were approached when they were about to buy food in supermarkets and hypermarkets for home consumption. The survey was administered using paper and provided an introduction about the aims of the study. The margin of error was below 3.54%, for a 95.5% confidence level (k = 2) under the principle of maximum in determination (*p* = *q* = 0.5). “k” is a constant that depends on the assigned level of confidence. The confidence level indicates the probability that the results of the research are correct. The binomial parameter, denoted *p*, is the probability of success; thus, the probability of failure is 1 − *p* which is often denoted *q*. Assigning success or failure to p is arbitrary and makes no difference. The sociodemographic characteristics of the respondents have been included in Table 1. Before the fieldwork, a preliminary questionnaire was administered to 40 lamb meat consumers to confirm that the survey questions were well designed and easily understandable.

### 2.2. Methods

Drawing on the framework of attributes proposed by Becker [39], the attributes considered to have the greatest effect on lamb meat consumers’ purchasing decisions were price, quality label and package appearance (search attributes), origin (credence attribute). and external appearance (amount, color or fat percentage (experience attribute). The color of meat depends on pigment content (mainly myoglobin), muscle protein and the proportion of fat infiltration [38]. Meat texture is perceived as the combination of tactile sensations resulting from the interaction of the senses with the physical and chemical properties of the meat. These sensations include density, toughness, plasticity, elasticity, consistency, moisture and the size of the meat particles. Of these, toughness is one of the primary determinants of meat quality for consumers [38]. The description of the attributes was not included in the survey. Additional information about the information of considered attributes is included in Table 2. Factors were selected considering previous studies addressing factors affecting lamb meat purchase decision-making [16,18,23,24,40] and the present authors’ experience Table 3. Additionally, to measure demographics and socioeconomics, respondents included information about their gender, age (in three established groups), highest level of education completed (in three groups), and household income (in five groups).

Respondents evaluated the importance of five key factors affecting their lamb meat buying decision on a 5-point Likert-type scale ranging from very unimportant (1) to very important (5).

The mean values obtained for the different attributes derived from the lamb meat consumers’ evaluations in 2004 and 2014 were compared using the *t*-test for independent samples. Following Schnettler et al. [41], we conducted a cluster analysis (hierarchical conglomerates) to identify principal consumer segments according to their preferences with regard to the lamb meat attributes that yielded significant differences (external appearance, origin, quality label, and price). Two different consumer segments were identified in 2004 and 2014. After the conglomerate analysis and with the aim to describe the differences between the reported segments, a *t*-test was used to examine responses about lamb meat attributes (external appearance, origin, quality label and price) and Pearson’s Chi-squared test was applied to discrete variables (age, highest level of education completed, and household income).

Results were analyzed using the Statistical Package for Social Sciences IBM SPSS v.22 (IBM Corp., Armonk, NY, USA).

## 3. Results and Discussion

Table 4 shows the relative importance of lamb meat attributes in 2004 and 2014. It can be observed that the most important attribute when purchasing in 2004 and 2014 is the external appearance of the product. However, differences appear in the order of the other attributes. In 2004, after external appearance, the most important attributes are the origin and the quality label in that order, while in 2014 consumers attached more importance to price than to attributes related to the origin or the form of production (origin and quality label).

A significant increase was observed in the perceived importance of price when purchasing lamb meat over the ten-year period. In this regard, the reduction of the frequency of consumption, together with the greater importance of price, limits the profit that lamb meat producers can obtain from their production. Consumers buy less lamb meat while also demanding lower prices when purchasing. Consumers are usually more willing to pay a higher price for products they do not consumer on a daily basis. In this regard, Cholette and Castaldi [42] affirm that wines intended for special occasions are likely to be more expensive than bottles bought for everyday consumption. However, in the case of lamb meat, the study of Bernabéu, Rabadán, El Orche, and Díaz [16] showed that occasional and regular consumers of lamb meat gave the same importance to lamb meat price when purchasing.

**Figure 1:**
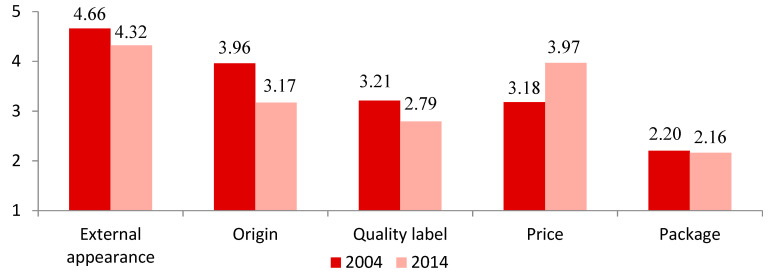
Importance of lamb meat attributes by consumer segment in 2004 and 2014. 5-point Likert-type scale ranging from very unimportant (1) to very important (5).

As can be seen in Figure 1, external appearance was the most important attribute for consumers in both 2004 and 2014 and the least important at both moments was the packing. The difference in lamb meat consumers’ preferences between 2004 and 2014 lies in the significance attached to price, with this being the fourth most important in 2004 but the second in importance in 2014. This change in the level of importance is probably due to the financial crisis affecting Spain between 2007 and 2017, given that, as reported in previous studies on periods of economic crisis, consumers are more sensitive to price [29,36,37]. A decrease in the quantities of food consumed and in the food spending because of the crisis were reported in Spain [43] with similar results in Greece [36]. In other severely affected countries, such as Portugal, it was observed an increase in the demand for generic products and cheaper brands [37].

It is also worth noting the decline in the importance attached to origin and quality seals, which have traditionally been used as a guarantee of the quality and safety of a product, ensuring its proper traceability [20,23]. It appears that, in the case of lamb, the demand for information and guarantees of the traceability and authenticity of the meat declines in times of economic crises, while the importance of the price attribute increases.

It seems paradoxical that given the growing concern for food safety, the packing attribute is only fifth in the order of importance among lamb meat consumers, with similar results in both 2004 and 2014, since packing serves as a container, protecting the meat from physical and chemical deterioration [44] and environmental contamination [45], and favoring longer shelf life as a result of various strategies, such as controlling temperature and moistness, adding different products [46] and oxygen removal, or a combination of these. Packing is thus a key element in the guarantee of quality and food safety [47].

This question is arguably due to lamb consumers focusing more on packing as a means of containing rather than protecting, especially in times of economic crisis, when the increase in the importance given to the price attribute plays a key role. The packing attribute may well have greater importance for consumers in an economic recession generated by a health crisis, as a guarantee of safety against the development of harmful micro-organisms [45].

Attending to consumption frequency, a significant reduction has been identified in the frequency of lamb meat consumption. While in 2004, the percentage of daily lamb meat consumption was 20.7% and monthly consumption was 63.3%, in 2014, daily and monthly consumption had fallen to 0.5% and 29.8%, respectively. It is worth noting that occasional lamb consumption increased between 2004 and 2014, perhaps as a result of the higher price of this meat relative to possible substitutes, its aspect, and also its traditional association with consumption on special occasions or festivities, as suggested by Blasco, et al. [48], all within a setting of reduced lamb meat consumption in the EU [1,2,3,4,8]. The results corroborate those of Díaz-Méndez and García-Espejo [34], who concluded that, in times of crisis, high-priced products and those for which medical guidelines recommend reduced intake (as is the case of lamb meat) suffer a notable reduction in consumption. Some other factors, such as the shifting from the consumption of red meat towards healthier white meat [6,7] can be also partially responsible for this reduction in the consumption of lamb meat.

The data obtained for 2004 yielded two significant segments of consumers according to their preferences with respect to the lamb meat attributes (Table 5).

In 2004, Segment 1 mainly valued the external aspects of the meat but also extrinsic quality aspects such as the origin and quality labels. The low importance they attached to the price of lamb meat when purchasing may be the result of their low frequency of consumption. Up to 85% of consumers in this segment purchase lamb meat once a month or less. As they only purchase lamb meat for special occasions, they buy high quality lamb meat, taking the origin and quality labels into consideration but paying little attention to price. Consumers in this segment are largely aged above 49 (up to 23.9% of them) compared to the 15.8% of consumers in Segment 2 who are on the same age range. They also have an average income slightly higher than the reported for the other segment, as up to 53.8% of them have an income of more than 1501 €. Regarding education, most of them had primary education and only 17.5% had university education.

Segment 2 is composed of a small group of consumers with a higher frequency of lamb meat consumption. Up to 28.9% of these individuals eat lamb meat on a daily basis. Due to this higher intake, they pay more attention to price when purchasing lamb and pay less attention to origin or quality labels. This consumer segment mainly consists of young people, where 23.7% of them were younger than 24 years old. Regarding education, most of them had primary education and only 21.1% of them had university education. Up to 60.6% of them had an average income ranging from 1500 € to 2100 €.

In contrast to the lamb purchasing segmentation observed in 2004, in 2014, the consumers segments showed no significant difference in the importance attributed to price (Table 6). For the two segments identified, the price is a crucial attribute. However, differences remain if the importance attributed to price is compared to the attitudes towards the other attributes. For Segment 2, the price is the third most important attribute, while it is the most important attribute for consumers in Segment 1.

In 2014, Segment 2 considered external appearance to be the most important attribute of lamb meat, followed by quality labels and price, with origin as the least important, while Segment 1 was guided most by price. This conflicts notably with the findings of previous studies in which the price attribute was one of those with least impact on meat consumers’ purchasing decisions [20,23].

It is worth noting also that lamb meat consumers value quality labels as an element that differentiates healthier meat with greater guarantees of safety, as reported in the previous literature [1,2,3,4,14,15,18].

Nonetheless, it is paradoxical that the origin of the lamb is the third and fourth most important attribute for consumers in Segments 1 and 2, respectively, when many previous studies have found origin to be the most appreciated attribute [16,23]. This might be due to the economic crisis or a disregard for the quality attributed to the origin of the product, leading to consumers basing their buying decisions more on the attribute of experience [48].

In addition, from a socioeconomic perspective, consumers in Segment 1 were mainly men over 49. In comparison, Segment 2 was mainly composed of young women. Regarding education, consumers on Segment 1 showed on average a lower level of education as only 40.7% of them had a university degree; this was 50.9% of consumers in Segment 2. The income was also slightly higher in consumers of Segment 2, possibly due to a higher educational background.

## 4. Conclusions

The main attribute valued by consumers in both situations under study (before and during the economic crisis) is the external appearance of the lamb, this being associated with the color of the meat, amount, and color of fat and texture.

The difference between the two moments of data collection lies in that before the crisis the price attribute was the fourth most important attribute in consumers’ purchasing decisions, while during the crisis this rose to second position, behind external appearance and ahead of origin, quality label and packing. In other words, the relative importance of the attributes forming consumers’ preferences changed in order to adapt to the situation of economic crisis.

In light of the results, we can conclude that crises lead consumers to reorganize the attributes that form their general preferences and purchasing decisions, with a specific impact on relatively high-priced products that are consumed less frequently, as might be the case of lamb meat.

To avoid or mitigate the drop in the frequency of consumption in periods of crisis, lamb meat producers and retailers could implement marketing campaigns in times of pre-crisis, aimed at younger individuals, informing about the good relationship between price and quality of their products, their food safety, healthy nutritional characteristics, and traditional, sustainable production based on rural environments.

The strength of the study relies on the amount of data collected that allowed us to evaluate the evolution of consumer preferences in time. This is even more interesting when some relevant shock, such as the economic crisis of 2008, hit society during that period. The weakness of the study relies on the limitation that appears to discriminate the impact of the economic crisis of any other reason that can affect consumer purchasing behavior. However, obtained results support the conclusions on previous studies about the increase on the importance of the price attribute under economic crisis scenarios.

There are two main limitations to this work. First, the surveys were only administered in specific locations while the results have been extrapolated to the country as a whole. Second, market research always has the weakness that there may be a difference between what consumer respondents say and what they actually do.

A possible future line of research would be to compare the projections derived from the economic crisis in 2014 with the 2020 public health crisis resulting from the COVID-19 pandemic, to determine similarities and differences and analyze variations in the relative importance of the attributes in the formation of lamb meat consumers’ preferences in two crisis with a very different origin. It would also be advisable to compare the results of this study with the results obtained from studies in other European countries to evaluate if preferences from consumers from different backgrounds change in the same direction.

## Figures and Tables

**Table 1:** Sociodemographic characteristics of lamb meat consumers in 2004 and 2014.

Variable	2004 Percentage (%)	2014 Percentage (%)
*Gender*		
Male	25.2	50.9
Female	74.8	49.1
*Age (years)*		
18–24	9.8	9.3
25–49	46.8	55.9
>49	33.4	35.7
*Education level*		
Elementary	47.3	17.9
Secondary	30.3	33.7
University	22.4	48.5
*Monthly income (€)*		
<900	25.6	18.3
900–1500	29.2	30.4
1501–2100	26.2	28.2
2101–3000	12.3	16.8
>3000	6.7	6.3

**Table 2:** Comparison of lamb meat consumer preferences in 2004 and 2014.

Lamb Meat Consumer Preferences	Year 2004 (*N* = 400)	Year 2014 (*N* = 400)
*Lamb meat attributes*		
External appearance	4.66 ^a^	4.32 ^b^
Origin	3.96 ^a^	3.17 ^b^
Quality label	3.21 ^a^	2.79 ^b^
Price	3.18 ^b^	3.97 ^a^
Package	2.20	2.16
*Lamb meat consumption frequency (%)*		
Daily	20.7	0.5
Once a week	13.5	11.5
Once a month	63.3	29.8
Occasionally	2.5	58.3

Different letters in the same row mean significant differences for lamb meat attributes (*p* < 0.05). Squared values for lamb meat consumption frequency are χ^2^ = 261.197, degrees of freedom = 3, *p* = 0.000.

**Table 3:** Lamb meat attributes evaluated by consumers in 2004 and 2014.

Lamb Meat Attributes	
External appearance	Set of external attributes that can be observed in meat purchase (e.g., amount, color or fat percentage)
Origin	The place where the lamb meat has been produced
Quality label	Label that indicates differentiated quality (e.g., PGI or PDO)
Price	Amount paid in euros (€)
Package	The way in which the lamb meat is wrapped or packed

Protected Geographical Indication: PGI; Protected Designation of Origin (PFO).

**Table 4:** Comparison of lamb meat consumer preferences in 2004 and 2014.

Lamb Meat Consumer Preferences	Year 2004 (*N* = 400)	Year 2014 (*N* = 400)
*Lamb meat attributes*		
External appearance	4.66 ^a^	4.32 ^b^
Origin	3.96 ^a^	3.17 ^b^
Quality label	3.21 ^a^	2.79 ^b^
Price	3.18 ^b^	3.97 ^a^
Package	2.20	2.16
*Lamb meat consumption frequency (%)*		
Daily	20.7	0.5
Once a week	13.5	11.5
Once a month	63.3	29.8
Occasionally	2.5	58.3

Different letters in the same row mean significant differences for lamb meat attributes (*p* < 0.05). Squared values for lamb meat consumption frequency are χ^2^ = 261.197, df = 3, *p* = 0.000.

**Table 5:** Consumer segmentation according to the evaluation of four lamb meat attributes in 2004.

Variables	Segment 1 (86.0%) ^1^	Segment 2 (14.0%) ^1^
*Lamb meat attributes*		
External appearance	4.70	4.68
Origin	4.35 ^a^	1.79 ^b^
Quality labels	3.43 ^a^	2.03 ^b^
Price	3.11 ^b^	3.71 ^a^
*Lamb meat consumption frequency (%)*		
Daily	19.2	28.9
Once a week	13.2	15.8
Once a month	85.0	52.6
Occasionally	2.6	2.6
*Socioeconomic characteristics (%)*		
*Gender*		
Male	25.2	26.3
Female	74.8	73.7
*Age (in years)*		
18–24	11.5	23.7
25–49	64.5	60.5
>49	23.9	15.8
*Education level*		
Elementary	49.1	42.1
Secondary	33.3	36.8
University	17.5	21.1
*Income*		
<900 €	14.1	15.8
900–1500 €	32.1	39.5
1501–2100 €	29.5	21.1
2101–300 €	16.2	15.8
>3000 €	8.1	7.9

^1^ Size of segment. Different letters in the same row mean significant differences for lamb meat attributes (*p* < 0.05). Chi-squared values for lamb meat consumption frequency and the socioeconomic variables are: consumption frequency, χ^2^ = 2.44, df = 3, *p* = 0.486; gender, χ^2^ = 0.021, df = 1, *p* = 0.885; age, χ^2^ = 4.676, df = 2, *p* = 0.096; education, χ^2^ = 0.682, df = 2, *p* = 0.711; income, χ^2^ = 1.44, df = 4, *p* = 0.838.

**Table 6:** Consumer segmentation according to the evaluation of four lamb meat attributes in 2014.

Variables	Segment 1 (28.0%) ^1^	Segment 2 (72.0%) ^1^
*Lamb meat attributes*		
External appearance	3.83 ^b^	4.51 ^a^
Origin	1.45 ^b^	3.83 ^a^
Quality labels	1.05 ^b^	4.48 ^a^
Price	3.86	4.01
*Lamb meat consumption frequency (%)*		
Daily	18.4	11.6
Once a week	28.6	30.3
Once a month	34.7	36.8
Occasionally	18.4	21.4
*Socioeconomic characteristics (%)*		
*Gender*		
Male	53.2	49.8
Female	46.8	50.2
*Age (in years)*		
18–24	4.6	11.1
25–49	46.7	57.7
>49	47.7	31.2
*Education level*		
Elementary	16.7	18.3
Secondary	42.6	30.8
University	40.7	50.9
*Income*		
<900 €	16.2	13.6
900–1500 €	33.3	27.2
1501–2100 €	27.6	28.3
2101–3000 €	14.3	19.6
>3000 €	8.6	11.3

^1^ Size of segment. Different letters in the same row mean significant differences for lamb meat attributes (*p* < 0.05). Chi-squared values for lamb meat consumption frequency and the socioeconomic variables are: consumption frequency, χ^2^ = 2.44, df = 3, *p* = 0.486; gender, χ^2^ = 0.360, df = 1, *p* = 0.548; age, χ^2^ = 15.94, df = 2, *p* = 0.003; education, χ^2^ = 4.946, df = 2, *p* = 0.084; income, χ^2^ = 3.079, df = 4, *p* = 0.545.
